# A Short Report: Self-Perceptions of Victimhood Among Women in Sex Trafficking Specialty Court Programming

**DOI:** 10.1007/s12103-026-09908-0

**Published:** 2026-04-02

**Authors:** Jane Mahon, Leah C. Butler, Gwen Jackson, Teresa C. Kulig, Aaron Murnan

**Affiliations:** 1 School of Criminal Justice, University of Cincinnati, 660 Teachers-Dyer Complex, PO Box 210389, Cincinnati, OH 45221-0389 USA; 2 Franklin County Municipal Court, Creating Autonomy Through Collaborative Healing (CATCH) Specialty Docket Court Program, Columbus, OH 43215 USA; 3 School of Criminology and Criminal Justice, University of Nebraska at Omaha, Omaha, NE USA; 4 The Ohio State University, Martha S. Pitzer Center for Women, Children and Youth, College of Nursing, Columbus, OH 43210 USA

**Keywords:** Sex trafficking specialty court, Victim, Survivor, Sex trafficking

## Abstract

While many women involved in the sex trade meet the legal criteria to be considered a sex trafficking victim, engagement in sex trafficking specialty court programs remain low. Although these programs offer benefits to participants, barriers impede engagement. One reason for low engagement may be that some women do not self-identify as a “trafficking victim,” and therefore do not see the program as being meant for them. To investigate this potential barrier, the current study was completed in partnership with three sex trafficking specialty court programs and one non-profit in Ohio. We conducted a cross-sectional, retrospective survey of 74 women to explore their self-perceptions of victimhood before and after entering the court program. These findings stand to educate criminal justice actors on how women with experiences of trafficking view and label their experiences over time. By better understanding the experiences and self-perceptions of those who have experienced trafficking, these programs can potentially help more individuals benefit from their resources who would otherwise be left out.

The United States has prioritized initiatives aimed at identifying sex trafficking victims and connecting them to resources (Clawson & Dutch, 2008). Based on federal law, sex trafficking involves experiencing force, fraud, or coercion when exchanging sexual services for something of value or doing so as a minor (Victims of Trafficking and Violence Protection Act of, 2000). Criminal justice actors are increasingly important for identifying trafficking behaviors as individuals with experiences of sex trafficking are often involved in other criminal behaviors, such as substance use, sex trading, or theft (Williams & Frederick, 2009). As a result, more than 30 sex trafficking specialty court (STSC) programs have been established across 10 states with the goal of improving identification of and access to services among those who have experienced trafficking (Kulig & Butler, 2019).

Although there is no universal operational procedure (Center for Court Innovation, 2015), these STSC programs are largely designed to redirect people who have experienced sex trafficking from traditional punishment to supportive health promotion services. After initial arrest, individuals can be connected to a STSC through (a) prostitution related charges (California Courts, 2014), (b) criminal justice actors identifying individuals as at-risk or actual victims of trafficking (Appleton, 2017), (c) trafficking screening tools (Liles et al., 2016), or (d) individuals applying for program entry (Kulig & Butler, 2019). Once enrolled, STSCs provide trauma-informed services aimed at addressing root problems underlying criminal recidivism and preventing future victimization (Appleton, 2017; Bell, 2016; Galindo, 2015).

These programs are continuing to emerge and have demonstrated promise (Kulig & Butler, 2019; Miner-Romanoff, 2017); however, identifying and engaging individuals to participate remains challenging. Although many women in the sex trade meet criteria for sex trafficking victimization, they are often not recognized as victims by law enforcement, service providers, or themselves. This may be due to the lack of universal screening tools (Macy et al., 2023) and difficulties discerning experiences of force, fraud, and coercion within sex trade involvement (Barrick et al., 2021). Further, individuals who experience sexual violence and sex trafficking frequently do not self-identify as a victim (e.g., Fohring, 2018; Marsil & McNamara, 2016). This is problematic as individuals who experience trafficking, but fail to identify as a victim, are unlikely to engage with beneficial services (Johnson & LaPlante, 2024; Kilimnik & Meston, 2019), and are at risk for future exploitation.

In this context, self-perceptions of victimhood may substantially impact someone’s engagement in STSCs designed to address the unique needs of those who have been trafficked (Moton, 2025). Yet, to our knowledge, only one study has explicitly assessed self-perceptions of victimhood among STSC participants (Moton, 2025). The court participants interviewed for this study varied to the extent to which they identified with and defined the concept “victim.” About one-third of participants rejected the victim label in its entirety. These findings suggest that self-identification is nuanced and individualized (Moton, 2025).

Understanding how individuals view their experiences are critical for STSCs to develop strategies to better identify and engage those who have experienced sex trafficking regardless of whether they see themselves as “victims.” Thus, this study builds off Moton (2025) by identifying broad trends among 74 STSC participants’ self-perceptions of victimhood and whether these perceptions change as they engage in STSCs.

## Methods

### Procedures and Participants

This study’s participants were recruited from three STSCs and one non-profit that serves individuals with experiences of trafficking between January and July 2025. All three STSC programs primarily serve individuals who live in Ohio, are over the age of 18, and identify as women. The non-profit provides wrap around services for former participants of a STSC included in this study. The research team shared study details with program staff, who then shared details with prospective participants. Those who expressed interest were then connected with the study team. To be eligible, prospective participants needed to: (1) be at least 18 years old, and (2) currently or previously participated in a STSC program. The study was approved by the University of Cincinnati's Institutional Review Board (ID#: 2025-0007).

Once the study team confirmed eligibility, study details, confidentiality, and the voluntary nature of the study were discussed with prospective participants. Participants were reassured that they would face no penalty if they chose not to participate or if they withdrew mid-survey. A waiver of written consent was used to protect confidentiality. Once informed consent was obtained, participants filled out a 20-minute survey. The survey included questions on sex trafficking experiences, self-perceptions of experiences and victimhood, and demographic information. Upon completion, participants received a $20 Visa gift card. Of the 74 participants who completed the survey, 61 participants were actively enrolled in a STSC while the remaining 13 participants had previous experience but were no longer enrolled in a STSC.

### Measures and Analyses

Measures of demographic characteristics include participant race, ethnicity, biological sex, gender, sexual orientation, and education status. Sex trafficking was measured in two ways. First, participants were asked about 12 *sex trafficking experiences after the age of 18* which captured force, fraud, and coercion (per federal legal criteria) (0 = did not experience; 1 = experienced) (see Kulig & Butler, 2024). Second, participants were also asked whether they engaged in the *sex trade as a minor* (0 = did not engage as a minor; 1 = engaged as a minor) (See Table 1). If participants reported any experiences of sex trafficking, including engaging in the sex trade as a minor, they were considered as having *experienced sex trafficking* (0 = did not experience sex trafficking; 1 = experienced sex trafficking).

**Table 1:** Sex trafficking experiences (*N* = 74)

Sex Trafficking Measures	Experienced % (*n*)	
**Minor Sex Trafficking**		
Engaged in the sex trade before age 18	98.6% (*n* = 73)	
**Adult Sex Trafficking**		
***To get you to exchange a sexual act for something of value after 18 years of age,*** ***did anyone...***		
**Force**	93.2% (*n* = 69)	
Actually punch, kick, slap, choke, burn, sexually assault, physically restrain, or otherwise physically harm you or someone you know	78.4% (*n* = 58)	
Monitor or control where you went	82.4% (*n* = 61)	
Restrict or control who you could speak with	78.4% (*n* = 58)	
**Fraud**	94.6% (*n* = 70)	
Tell you a lie	89.2% (*n* = 66)	
Make false promises to you about employment, money, or working conditions	72.3% (*n* = 54)	
Make false promises to you about love, marriage, or a better life	78.4% (*n* = 58)	
**Coercion**	94.6% (*n* = 70)	
Threaten to punch, kick, slap, choke, burn, sexually assault, physically restrain, or otherwise physically harm you or someone you know	82.4% (*n* = 61)	
Threaten to call legal authorities on you or someone you know	55.4% (*n* = 41)	
Threaten to withhold food, water, sleep, or medical care from you or someone you know	56.8% (*n* = 42)	
Threaten or actually take your personal documents (for example: identification, passport, social security card)	37.8% (*n* = 28)	
Threaten to share personal information or photos of you	62.2% (*n* = 46)	
Make you pay back a financial debt or loan by engaging in a sexual act	70.3% (*n* = 52)	

S*elf-perception of victimhood before court programming* was measured as a dichotomous variable where participants were asked whether they identified as a victim or survivor of sex trafficking *before* court programming (0 = did not identify as a victim; 1 = identified as a victim). *Self-perception of victimhood since court programming* was measured as a dichotomous variable where participants were asked whether they identified as a victim or survivor of sex trafficking *since starting* court programming (0 = did not identify as a victim; 1 = identified as a victim).

Given the exploratory nature of the study, descriptive statistics were used to identify broad trends. We begin by describing the makeup of the sample in terms of demographics and experiences in the sex trade, followed by reporting the trends in sex trafficking victim identification.

## Results

Among the 74 participants, the majority were White (60.8%, *n* = 45), followed by Black or African American (31.1%, *n* = 23), then another racial identity (8.1%, *n* = 6). Only 4.1% (*n* = 3) participants identified as Hispanic, Latino, or Spanish origin. All participants identified their biological sex as female (*n* = 74) and the majority identified as women (97.3%, *n* = 72). Most participants identified as heterosexual (62.2%, *n* = 46). In terms of education, 32.4% (*n* = 24) reported not completing high school requirements, 31.1% (*n* = 23) reported earning a GED or high school diploma, and 36.5% (*n* = 27) pursued education beyond high school.

All participants met the legal criteria for experiencing sex trafficking based on the federal legal definition (*n* = 74). Of the participants, 98.6% (*n* = 73) indicated experiencing force, fraud, and/or coercion after the age of 18, and 98.6% (*n* = 73) engaged in the sex trade as a minor. Of those who were trafficked as an adult, 93.2% (*n* = 69) experienced force, 94.6% (*n* = 70) experienced fraud, and 94.6% (*n* = 70) experienced coercion (see Table 1).

**Fig. 1:**
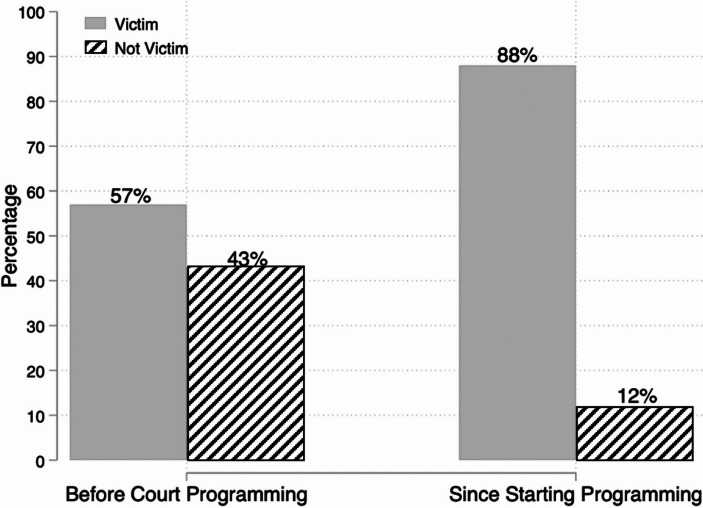
Victim identification before and since starting court programming (*N* = 74)

Before court programming, 43.2% (*n* = 32) of participants did not see themselves as a victim or survivor of sex trafficking. This number dropped to 12.2% (*n* = 9) since court program participation. See Fig. 1 for a comparison.

## Discussion

While some research suggests that women’s reluctance to self-identify as a “victim” impedes victim identification (Chaffee & English, 2015; Dank et al., 2015), only one study has explored self-perceptions of victimhood among STSC participants (Moton, 2025). This study expands off Moton’s (2025) work by providing a descriptive overview of 74 women who have been involved in a STSC regarding (1) sex trafficking experiences, (2) self-perceptions of victimhood, and (3) whether these perceptions change after STSC participation. These analyses have three key implications.

First, all participants met legal criteria for experiencing sex trafficking. Of the participants, 98.6% (*n* = 73) were trafficked as a minor and 98.6% (*n* = 73) experienced force, fraud, and/or coercion as an adult. Although participants reported various experiences of trafficking, one may wonder how these experiences may influence self-perceptions of victimhood. Future research should explore whether experiences with specific trafficking behaviors (i.e., force, fraud, or coercion), relationships with trafficker (e.g., family member, boyfriend), and familial norms regarding abuse shape self-perceptions of victimhood. If such experiences do not align with a victim’s perception of what “counts” as victimization, they may be less likely to be identified as victims by service providers or to choose to engage in STSCs or other services (Mason et al., 2024; Marcinkowski et al., 2022).

Second, despite all participants meeting legal criteria for sex trafficking victimization, 43% (*n* = 32) of participants did not identify as a survivor or victim before court programming. Research shows that those who do not identify as a survivor are less likely to seek and engage with formal help (Johnson & LaPlante, 2024). Thus, it is imperative that service providers recognize and identify trafficking behaviors to help facilitate connections to services regardless of individuals’ self-identification. Additionally, service providers should use more inclusive language beyond “sex trafficking victim” so that women who do not identify as such still feel included and open to engaging with STSC resources.

Third, self-perceptions of victimhood were shown to change over time. Thirty one percent of participants changed their self-perception to identify as a victim of sex trafficking since starting court programming. The question remains as to *why* participants changed their perceptions. Research suggests that sex trade participation is commonplace within some family environments, inclusive of interpersonal violence and parental substance use, as well as among those who have associations with others who trade sex (Choi, 2015; Cobbina & Oselin, 2011). Education on sex trafficking from the STSC may have helped participants recognize how their experiences align with the legal criteria of experiencing sex trafficking. Conversely, participants may have acclimated to the courtroom environment, normalizing sex trafficking victimhood and increasing self-identification. Future research should explore the mechanisms behind changes in self-identification and how these changes influence engagement with STSC services.

Several limitations should be noted. First, all participants had been identified as experiencing sex trafficking by criminal justice actors at some point before the survey. Second, the survey captured retrospective perceptions of victimhood before and after STSC participation rather than measuring their perceptions in real time during each of these periods. Third, this report focuses on responses to closed-ended questions; future research should use open-ended responses and interviews to capture reasons for victim identification as well as why identification may change overtime. Lastly, the sample included 74 women, limiting power for statistical analyses.

## Conclusion

Although all STSC participants met the legal criteria for experiencing sex trafficking, 43% of participants did not identify as a victim or survivor of sex trafficking before STSC programming. This number dropped to 12% since program participation. These findings highlight the complexity of self-perceptions of victimhood among women who have experienced trafficking. In this context, STSC may provide a safe environment for women to process and recognize their own victimization. Understanding the nuanced and complex nature of perceptions related to victimhood and sex trafficking is critical to best inform and improve engagement strategies for STSC by aligning program eligibility and recruitment approaches with women’s perceptions of their victimhood upon entering the criminal justice system.

## Data Availability

Data and materials are available from the corresponding author upon reasonable request.
